# Independent Left Ventricular Morphometric Atlases Show Consistent Relationships with Cardiovascular Risk Factors: A UK Biobank Study

**DOI:** 10.1038/s41598-018-37916-6

**Published:** 2019-02-04

**Authors:** Kathleen Gilbert, Wenjia Bai, Charlene Mauger, Pau Medrano-Gracia, Avan Suinesiaputra, Aaron M. Lee, Mihir M. Sanghvi, Nay Aung, Stefan K. Piechnik, Stefan Neubauer, Steffen E. Petersen, Daniel Rueckert, Alistair A. Young

**Affiliations:** 1 Auckland Bioengineering Institute, University of Auckland, Auckland, New Zealand; 2 Biomedical Image Analysis Group, Department of Computing, Imperial College London, London, UK; 3 Department of Anatomy and Medical Imaging, University of Auckland, Auckland, New Zealand; 4 William Harvey Research Institute, NIHR Barts Biomedical Research Centre, Queen Mary University of London, Charterhouse Square, London, EC1M 6BQ UK; 5 Barts Heart Centre, St Bartholomew’s Hospital, Barts Health NHS Trust, West Smithfield, London, UK; 6 Oxford NIHR Biomedical Research Centre, Division of Cardiovascular Medicine, Radcliffe Department of Medicine, University of Oxford, Oxford, UK; 7 Department of Biomedical Engineering, King’s College London, London, UK

## Abstract

Left ventricular (LV) mass and volume are important indicators of clinical and pre-clinical disease processes. However, much of the shape information present in modern imaging examinations is currently ignored. Morphometric atlases enable precise quantification of shape and function, but there has been no objective comparison of different atlases in the same cohort. We compared two independent LV atlases using MRI scans of 4547 UK Biobank participants: (i) a volume atlas derived by automatic non-rigid registration of image volumes to a common template, and (ii) a surface atlas derived from manually drawn epicardial and endocardial surface contours. The strength of associations between atlas principal components and cardiovascular risk factors (smoking, diabetes, high blood pressure, high cholesterol and angina) were quantified with logistic regression models and five-fold cross validation, using area under the ROC curve (AUC) and Akaike Information Criterion (AIC) metrics. Both atlases exhibited similar principal components, showed similar relationships with risk factors, and had stronger associations (higher AUC and lower AIC) than a reference model based on LV mass and volume, for all risk factors (DeLong p < 0.05). Morphometric variations associated with each risk factor could be quantified and visualized and were similar between atlases. UK Biobank LV shape atlases are robust to construction method and show stronger relationships with cardiovascular risk factors than mass and volume.

## Introduction

Left ventricular (LV) morphology and function is important for the evaluation of cardiovascular disease. Changes in shape, known as remodeling, can manifest as changes in LV mass, volume, sphericity, wall thickness, and other shape indices, due to clinical and pre-clinical disease processes. Previous studies have shown the importance of remodeling in the evaluation of 10-year survival rates after a myocardial infarction^1–3^. Pre-clinical remodeling also occurs in asymptomatic individuals prior to the establishment of clinical disease, in response to exposure to risk factors and genetic interactions^4^. However, current shape measures of LV mass and volume ignore most of the shape information available in modern medical imaging examinations. The UK Biobank employed cardiac magnetic resonance (CMR) imaging to examine the pre-clinical determinants of cardiac disease^5,6^. This large-scale cohort study has enabled investigation of reference characteristics in healthy participants^7^, and mechanistic relationships with cardiovascular risk factors^8^.

Atlases of the LV have recently been employed to produce statistical shape models, giving highly detailed morphometric information in a standardized coordinate space, suitable for large cohort studies^9–11^. Compared with the American Heart Association 17-myocardial segment model^12^, atlas-based analyses represent morphology at high spatial resolution, enabling quantification of multidimensional statistical information at each point in the atlas^13^. Changes in LV morphometry have been demonstrated in healthy adults who were born prematurely^14^, volunteers with titin-truncating variants^15^, genetic mutations affecting LV mass^16^, higher fat mass^17^, higher blood pressure^13^, and smoking and other risk factors in the Multi-Ethnic Study of Atherosclerosis (MESA)^9^. Atlas based methods have also been used to quantify remodeling patterns in patients with myocardial infarction^18^, shape features associated with response after cardiac resynchronization therapy^19^, and impairment of function in congenital heart disease^20^.

However, atlas-based shape measures may be influenced by the methods used in their construction, and this may affect the resulting shape analyses. Two different types of atlas have been derived to date, using either volume image registration^10,13,15–17^ or surface registration^9,14,18–20^ methods. The key difference between these approaches is in the non-rigid registration techniques used to map the anatomy of each patient’s heart into a common coordinate system. Volumetric image registration methods utilize image intensity features to compute the mapping of each case onto the common space. Conversely, surface registration methods use knowledge of the boundaries of the heart in the registration process. Each type of atlas may therefore result in different LV shape characteristics. To date, there has been no objective comparison of different types of atlas in the same cohort.

Here, we describe the construction of two types of atlas, derived using volume and surface registration methods respectively, from 4,547 participants of the CMR extension to UK Biobank. By comparing morphometric indices between atlases, we investigated the extent to which results were dependent on the method used to construct the atlas. We also examined whether the shape atlases provided stronger relationships with known cardiovascular risk factors, in comparison with the standard indices of LV mass and volume. We also compared morphometric risk factor scores and morphometric shape variations from both atlases to characterize the associations between LV shape and cardiovascular risk factors.

## Results

**Figure 1:**
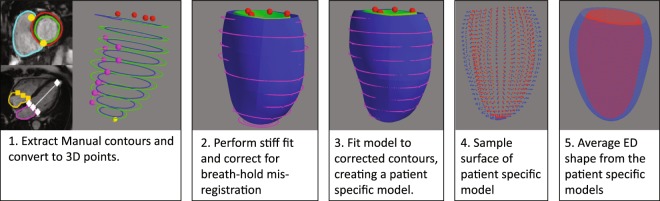
Surface atlas construction. Left to right: Images to average shape model.

**Figure 2:**
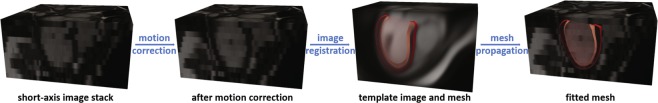
Volume atlas construction. Left to right: Images to shape model.

Of the first 5,065 CMR UK Biobank imaging extension participants, 4,547 common cases could be used to construct volume and surface atlases. The remaining 518 cases had missing information required for one or other of the atlases (either missing images or contours). Table 1 shows the participant characteristics. The surface atlas construction process is shown in Fig. 1, and the volume atlas construction process is shown in Fig. 2. Of the 4,547 cases in both atlases, 751 were identified in the reference healthy cohort with no risk factors^7^.

**Table 1:** Participant characteristics for those cases in both atlases (n = 4547).

Age (years)	62 ± 8
Sex (male)	2153 (47%)
Height (cm)	170 ± 9
Weight (kg)	76 ± 15
Body surface area (m^2^)	1.85 ± 0.21
Systolic blood pressure (mmHg)	139 ± 19
Diastolic blood pressure (mmHg)	79 ± 11
Heart Rate (bpm)	68 ± 11
High blood pressure	1183 (26%)
Smoking (never)	2688 (59%)
Smoking (previous)	1552 (34%)
Smoking (current)	296 (7%)
Diabetes	235 (5%)
Angina	104 (2%)
Asthma	493 (11%)
High Cholesterol	1183 (26%)

Values are given as mean ± standard deviation for continuous variables, and count (%) for categorical variables.

**Figure 3:**
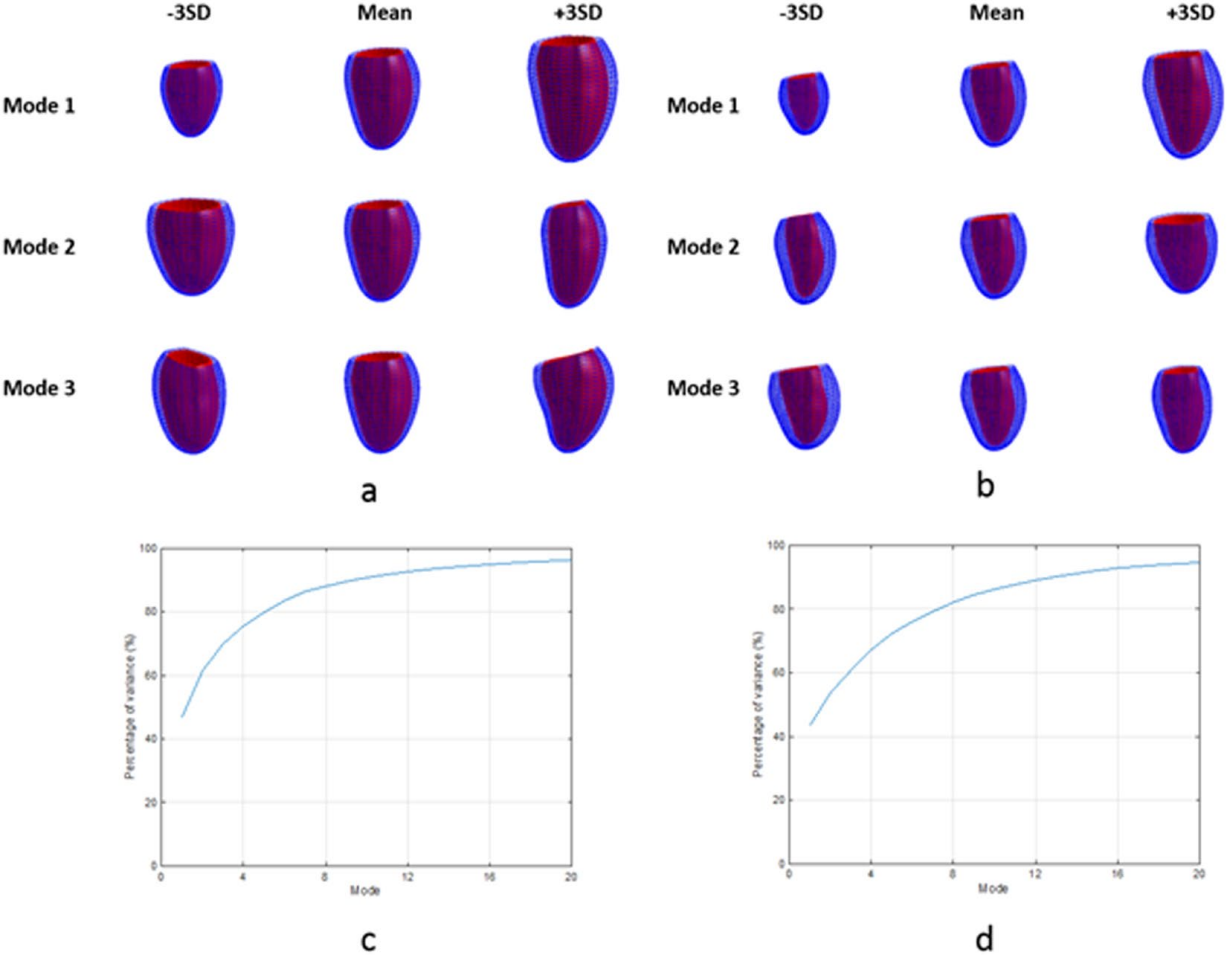
Principal component analysis results for the surface atlas. (**a**) ED first three principal components; (**b**) ES first three principal components; (**c**) ED % variance explained for the first 20 modes; (**d**) ES % variance explained for the first 20 modes. The viewpoint is from the septum with the inferior wall on the left.

Figure 3 shows the first three principal component shape modes describing the most variation in the cohort, for the surface atlas at both end-diastole (ED) and end-systole (ES). The graphs show the cumulative amount of variance explained by each principal component mode for the first 20 modes in each atlas. In the surface atlas, the first principal component (explaining over 40% of the total variance) was associated with LV size for both ED and ES. The second principal component was associated with LV height to width ratio, or sphericity. The third principal component was associated with the mitral valve plane orientation.

**Figure 4:**
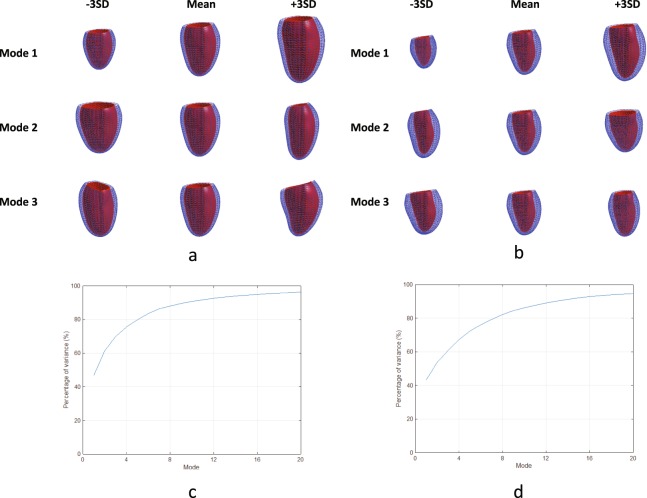
Principal component analysis results for the volume atlas. (**a**) ED first three principal components; (**b**) ES first three principal components; (**c**) ED % variance explained for the first 20 modes; (**d**) ES % variance explained for the first 20 modes. The viewpoint is from the septum with the inferior wall on the left.

Figure 4 shows the first three principal component shape modes of the volume atlas, together with graphs of cumulative percentage variance explained for the first 20 modes. Similar to the surface atlas, the first principal component was associated with LV size, the second with sphericity and the third with mitral valve plane orientation. Each mode explained a similar proportion of the total variance as the corresponding surface atlas mode.

**Figure 5:**
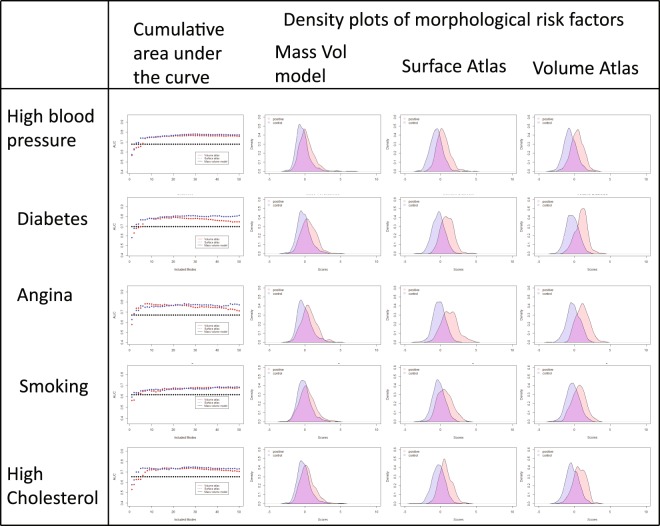
Cumulative area under the curve with increasing numbers of modes included and density of morphometric risk factor scores. Scores for the reference (healthy) cohort are shown in blue and those for risk factor positive cases are shown in orange.

Table 2 shows the strength of relationships between the risk factors and shape, using logistic regression analysis with five-fold cross validation. Each risk factor was treated as the dependent variable in the logistic regression analysis, with 1 for participants positive for the risk factor and 0 for the participants in the reference healthy cohort with no risk factors^7^. For each analysis, the first 20 scores from the ED principal component analysis and the first 20 scores from the ES principal component analysis were used as independent variables. For comparison, a similar logistic regression cross-validation analysis was also performed using the traditional measures of LV mass, ED and ES volume as the independent variables (termed “MassVol” in Table 2). Both volume and surface atlases gave stronger associations, i.e. larger AUC and lower AIC, between LV shape and all risk factors, compared to the MassVol model. Similar AUC (DeLong p = NS) and AIC values were found between the surface and volume atlases. Figure 5 shows the AUC for each risk factor as the number of principal component modes was increased from 1 to 50. Approximately three ED and ES modes were needed for the atlases to achieve comparable performance to the MassVol model. Performance was stable after about 7 modes.

**Table 2:** Five-fold cross-validated logistic regression analysis results for binomial categorical factors and LV shape (first 20 principal component modes from ED and ES).

	Volume Atlas	Surface Atlas	MassVol
High blood pressure	0.77*** (2157)	0.76*** (2143)	0.68 (2382)
Smoking	0.68* (1174)	0.68* (1156)	0.62 (1213)
Diabetes	0.80*** (857)	0.79*** (869)	0.70 (1001)
High cholesterol	0.73** (1124)	0.73** (1126)	0.65 (1224)
Angina	0.77* (551)	0.76* (528)	0.67 (607)

MassVol model includes LV mass, EDV and ESV as independent variables. Each cell has AUC (AIC). *P < 0.05, **P < 0.01, ***P < 0.001, DeLong’s test for differences in AUC from MassVol AUC.

**Figure 6:**
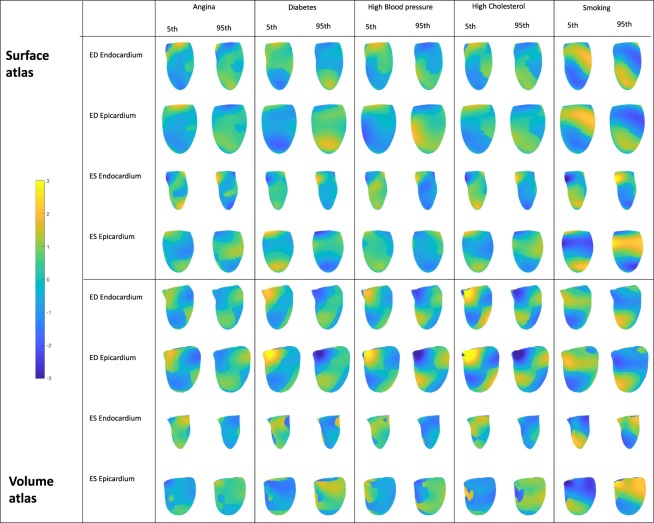
Morphometric risk factor shapes. The 9^th^ and 95^th^ percentile of the logistic regression models rendered at ED and ES. The average shapes were drawn with differences shown in the color scale yellow (outward surface movement) to blue (inward surface movement). View point is from the anterior, with septum on the left. Displacements are in mm.

Morphometric risk factor scores were calculated over the whole cohort using the logistic regression coefficients to combine component scores into a single z-score for each risk factor. Similar score distributions were found for the volume and surface atlases. Figure 5 shows density plots for the morphometric risk factor score associated with the risk factors. In each plot two patient groups are shown with the reference cohort (blue) and the risk factor positive cohort (orange). Both atlases had higher separation of scores between groups than those derived from the MassVol model for all risk factors. Figure 6 shows the 5^th^ and 95^th^ percentiles of the morphometric shape variation associated with each factor. The figure shows the variation in shape, as weighted by the model regression coefficients. Angina was associated with an overall outward displacement at ED. Diabetes was associated with a bulging at the apex. High blood pressure, high cholesterol, and smoking were associated with a septal outward displacement toward the apex but inward near the base.

## Discussion

The results of this study indicate that LV shape atlases show consistent relationships with cardiovascular risk factors, irrespective of the methodology used to derive the atlas. Two types of atlas were constructed from the same cohort of CMR examinations, performed as part of the UK Biobank imaging extension. The two atlases used different methods to calculate shape characteristics, the volume atlas being based on intensity differences in the MRI cine images, and the surface atlas being based on manual contours drawn on the epicardial and endocardial surfaces. However, both had similar principal component shape modes, similar principal components, and similar associations with cardiovascular risk factors. These associations were stronger than those with standard measures of LV mass and volume, in both atlases. These results suggest that shape features derived from these atlases are not severely impacted by methodology, but express real anatomical characteristics related to cardiovascular risk factors.

In a previous LV surface atlas study from other asymptomatic cohort (the Multi-Study of Atherosclerosis or MESA)^9^, we found similar shape modes. The shape mode explaining the most variation (first principal shape mode) was associated with LV size in both volume and surface atlases, as well as in the MESA atlas. The second principal shape mode, orthogonal to the first, describes LV sphericity in both UK Biobank atlases, and also in the MESA atlas^9^. Both LV size and sphericity are known to be associated with adverse events in patients with clinical disease and in largely healthy populations^1–4^.

Both atlases had stronger associations with risk factors than traditional measures of LV morphometry (mass and volumes). Similar associations were found between shape and risk factors regardless of atlas construction. In addition to providing information on the morphological changes associated with risk factors, these atlases can also be used to evaluate individual patients during longitudinal follow-up. For example, z-scores could be calculated for each visit, indicating where the patient ranks in relation to the UK Biobank population. A change in z-score towards the positive end would indicate a deterioration in LV shape over time. Morphometric risk factor scores can then be included in future studies of outcomes over time, for example in Cox models along with sex, body mass index and age, in comparison to the risk factors themselves.

Limitations of this study include the cross-sectional nature of the UK Biobank; however, as events are recorded into the future, it may be possible to determine the extent to which LV shape features can add to prediction of future events. Also, neither cholesterol nor glucose blood test data was available at the time of writing. Risk factors were self-reported and may suffer from subjective bias. Since the intention of the current study was to compare shape atlases, we did not correct shape scores for sex, age, body mass index, etc. In the future, these atlases could be used in conjunction with other factors in multivariate models to better understand shape changes in more targeted groups, such as those with different environmental or haemodynamic factors. Finally, the surface approach requires initial segmentation of the LV contours and landmarks; however, machine learning methods show promise to automatically provide these data^21^.

## Conclusions

Both volume and surface cardiac atlases show similar morphometric characteristics, suggesting that shape scores derived from LV atlases are robust and quantify real anatomical relationships with cardiovascular risk factors. Morphometric scores are more sensitive to detect differences in LV shape associated with cardiovascular risk factors than traditional measures of mass and volume.

## Methods

### Study Population

The UK Biobank has collected questionnaire data, physical measurements and biological samples from 500,000 individuals in the UK^22^. The imaging extension aims to obtain brain, heart, whole body composition, carotid artery, bone and joint imaging in 100,000 participants by 2022. Here, we assessed CMR examinations from the first 5,065 UK Biobank imaging extension participants. All participants gave written informed consent and the appropriate institutional review boards approved the study protocol (National Research Ethics Service North West 11/NW/0382). All research was performed in accordance with the relevant guidelines and regulations.

Similar to a recent analysis of relationships between cardiovascular risk factors and LV mass and volume in the same cohort^8^, we investigated the associations between multidimensional LV shape derived from the atlases, and each of the following risk factors: high blood pressure, smoking, diabetes, high cholesterol, and angina. High blood pressure, diabetes, high cholesterol and angina were taken from self-reported vascular/heart or non-cancer conditions or problems diagnosed by physician. Smoking was taken as current tobacco smokers. Participants positive for each risk factor were compared with a reference healthy cohort defined according to the criteria described in^7^. The reference cohort excluded all participants with known cardiovascular disease, hypertension, respiratory disease, diabetes, hyperlipidaemia, haematological disease, renal disease, rheumatological disease, malignancy, symptoms of chest pain or dyspnoea, age over 74 years old, current- or ex-tobacco smokers, those taking medication for diabetes, hyperlipidaemia or hypertension and those with BMI ≥ 30 kg/m^2^.

### Imaging Protocol

The CMR protocol has been described in detail previously^23^. Briefly, all imaging was performed on a wide bore 1.5 T scanner (MAGNETOM Aera, *syngo* MR D13A, Siemens Healthineers, Erlangen, Germany) using a phased-array cardiac coil. Retrospectively gated cine balanced steady-state free precession breath-hold acquisitions were performed in horizontal long axis, vertical long axis, left ventricular outflow tract orientations, and a short axis stack covering the left and right ventricles. Typical parameters were: TR/TE = 2.6/1.1 ms, flip angle 80°, GRAPPA factor 2, voxel size 1.8 × 1.8 × 8 mm³ (6 mm for long axis). The actual temporal resolution of 32 ms was interpolated to 50 phases per cardiac cycle (~20 ms). No signal or image filtering was applied besides distortion correction.

### Manual Analysis

The manual contouring process was performed in accordance with the Society of Cardiovascular Magnetic Resonance recommendations, as described in detail previously^7^. Briefly, short axis images were contoured at end-diastole (ED) and end-systole (ES) using cvi^42^ post-processing software (Version 5.1.1, Circle Cardiovascular Imaging Inc., Calgary, Canada), by eight readers in two core laboratories. The ED frame was selected as the first frame after detection of the R wave, and the ES frame was selected as the smallest LV blood pool area in the mid-ventricular slice. At both ED and ES, the most basal slice included had at least 50% of the LV blood pool surrounded by myocardium. Papillary muscles were included in the blood pool. Left atrial contours delineated on the two chamber and four chamber long axis slices, and right ventricular contours on the short axis slices, were also used in this study to delineate the extent of the LV and the location of the interventricular septum. Interobserver agreement in mass and volume estimates was excellent, with intra-class correlation coefficients of 0.88 and above^7^.

### Surface Atlas

The surface atlas was constructed using the method described by Medrano-Gracia *et al*.^9^. Briefly, a finite element shape model was fitted to the manual contours by least squares optimization. The extent of the LV was defined from landmarks on mitral valve (derived from the extent of the left atrium contour) and a LV apex point obtained from the cvi^42^ contour files. The septum was located using the insertions of the RV contour with the LV contour (calculated from the RV endocardial contour). After orienting the model according to the landmarks, the endocardial and epicardial surfaces were fitted to the landmarks and short axis contours by minimizing the distance between the surfaces and the contour points. Mis-registrations of the contours due to differences in the breath-hold position from slice to slice were automatically corrected by shifting the contours in-plane to match an initial stiff model fit^9^. Figure 1 illustrates the formation of the resulting atlas.

### Volume Atlas

The volume atlas was constructed using the method described by Bai *et al*.^10^. Briefly, a common template image space and myocardial mesh were used, which were previously derived from high-resolution 3D MR images [8]. Each short axis image stack was first corrected for breath-hold mis-registration using the same method as for the surface atlas construction. Each corrected image volume was then registered to the template space^10^ using non-rigid B-spline image registration^24^. For each case, the displacement field was stored to give a mapping from subject space to template space at each voxel. The template mesh was propagated to each subject using the inverse displacement map. Each subject mesh then had the same number of vertices as the template mesh. Figure 2 shows the construction of the resulting atlas.

### Statistics

For each atlas, two statistical shape models were generated, one at ED and the other at ES, using principal component analysis. This procedure calculated the principal modes of shape variation across the cohort, ordered by amount of variance explained^11^. Firstly, point clouds were generated on the epicardial and endocardial surfaces of the finite element model, or from the volume atlas myocardial mesh. Within each statistical shape model, the point clouds were aligned using the Procrustes method^25^, without scale correction (i.e. translation and rotation alignment only). Principal component shape modes were then calculated at ED and ES as described previously^9,10^. Each case could then be represented by a set of principal component scores, which represent the amount of each mode present in that case.

Associations between LV shape and risk factors were examined using logistic regression linear models. For each risk factor, a separate linear model was generated using that factor as a binary univariate dependent variable, and the principal component scores as the independent variables. The strength of the association between shape and risk factor was quantified using two metrics: (i) the area under the curve of the receiver operating characteristic (AUC) and (ii) the Akaike information criterion (AIC). The AUC is a measure of the overall performance of the logistic regression model and reflects the probability of correctly ranking any pair of positive/negative cases^26^. A value closer to 1.0 is indicative of a better model. The AIC is a measure of relative quality of the model, with lower values indicating better goodness of fit corrected for the independent variables^27^. To prevent overfitting, a five-fold cross validation scheme was employed in which the dataset was randomly divided into five groups, and the model trained on 4/5 and tested on the remaining 1/5 for each of the five groups. For comparison, logistic regression cross-validation models were also formed using ED volume, ES volume and LV mass as the independent variables, and the strengths of association compared with the shape atlases. Significant improvements in AUC were tested using DeLong’s test^28^.

Morphometric risk factor scores were derived from the logistic regression coefficients obtained for each risk factor, as follows. The logistic regression coefficients represent a linear combination of principal shape modes which best describe differences between the reference cohort and the risk factor positive participants. Therefore, a combined score was calculated by multiplying each principal component score by its corresponding logistic regression weight and summing over components. These scores were calculated for all participants and normalized into z-scores. These scores provide a simple way of quantifying shape characteristics for each case in relation to the population. Similarly, a morphometric risk factor shape variation could be calculated to visualize the shape change associated with the morphometric score.

Statistical analysis was performed using R (version 3.3.0) Statistical Software^29^ and the caret package^30^.

### Declarations

#### Ethics Approval and Consent to Participate

The appropriate institutional review boards approved the study protocol (National Research Ethics Service North West 11/NW/0382). All participants in this study gave written consent to participate as part of the UK Biobank recruitment process.

#### Consent for Publication

All participants in this study gave written consent to publish as part of the UK Biobank recruitment process.

## Data Availability

UK Biobank encourages and provides as wide access as possible to its data and samples for health-related research in the public interest by all bona fide researchers from the academic, charity, public, and commercial sectors, both in the UK and internationally, without preferential or exclusive access for any user. Data can be sought directly from UK Biobank via online application at http:// www.ukbiobank.ac.uk/register-apply/. The atlases generated in this paper are available from UK Biobank, and statistical shape models from www.cardiacatlas.org.
